# Inbreeding depression of reproductive traits in Japanese Black cattle using genomic information

**DOI:** 10.5713/ab.25.0113

**Published:** 2025-09-01

**Authors:** Takayuki Ibi, Souma Kouno

**Affiliations:** 1 Graduate School of Environmental, Life and Natural Science, Okayama University, Okayama, Japan

**Keywords:** Genomic Information, Inbreeding Depression, Japanese Black

## Abstract

**Objective:**

The objective of this study was to evaluate genomic inbreeding in Japanese Black cattle and its effects on reproductive traits.

**Methods:**

The study analyzed reproductive records and single nucleotide polymorphism (SNP) data from Japanese Black cattle born between 2001 and 2005, resulting in 8,553 records from large farms. Genomic inbreeding was assessed using SNP data from 782 animals. Key reproductive traits analyzed included age at first calving (AFC) and calf production index (CPI). Statistical models were employed to estimate fixed effects and inbreeding depression.

**Results:**

Findings indicated that genomic inbreeding levels were generally higher than traditional inbreeding coefficients. Significant inbreeding depression was observed in CPI but not in AFC. Specific chromosomes (12, 15, 27 for CPI and 4 for AFC) showed significant associations with inbreeding depression. Key genes related to reproduction were identified, particularly on chromosome 15.

**Conclusion:**

The study concludes that genomic inbreeding effectively detects inbreeding depression related to reproductive abilities in Japanese Black cattle. The results highlight the importance of genomic assessment in breeding practices to enhance reproductive performance while maintaining genetic diversity in the breed.

## INTRODUCTION

Japanese Black cattle are the most common beef cattle breed in Japan, known for their excellent meat quality. Their meat is especially famous for its high degree of marbling, which is the characteristic trait of Japanese Black cattle.

The meat quality of Japanese Black cattle has been improved to differentiate them from foreign breeds; however, in recent years, improvements in reproductive traits, such as age at first birth and calving interval, have been promoted. Beef production is closely related to production efficiency, as a one-month extension in the cow conception would increase maintenance costs by one month. Therefore, reproductive traits are important for farmers, and the government has established livestock improvement goals. However, the improvement of reproductive traits was stagnant and lower than that of carcass traits, due to the reduced genetic diversity from high inbreeding to improve carcass traits, and the lower heritability of reproductive traits compared with that of carcass traits.

The stagnation in the improvement of reproductive traits might be due to an increase in the inbreeding coefficient. Since the establishment of a system for breeding value evaluation using artificial insemination technology and the BLUP method, rapid improvements have been made [1]; however, the demand for specific breeds of bulls and their blood relatives has increased. Inbreeding coefficients have increased since 1990. Inbreeding is known to cause inbreeding depression [2].

The inbreeding coefficient has been used as an indicator of inbreeding frequency. However, some drawbacks of the inbreeding coefficient have been identified, including the fact that it is a probability and cannot be measured accurately when pedigree information is missing or incomplete. In recent years, new inbreeding indices have been developed to solve these problems. They use molecular genetic information such as single nucleotide polymorphisms (SNPs). The run of homozygosity (ROH) has been reported to be the most effective of them [3]. The ROH is a region where a certain number of homozygous SNPs are present. The proportion of ROH regions in the whole genome was defined as the proportion of homologous homozygotes in the genome (genomic inbreeding). The degree of genomic inbreeding is an actual value obtained by directly observing the genomic information, and it is not affected by the presence or absence of pedigree information. Moreover, it is possible to measure the degree of inbreeding for each chromosome or each specific region using the degree of genomic inbreeding, which is impossible while using the inbreeding coefficient. Studies on the degree of genomic inbreeding have been conducted overseas for several years; however, most of them have targeted dairy cattle, and there are few studies on beef cattle. In addition, the Japanese Black cattle breed is unique to Japan. Thus, the meat quality and reproductive ability of this breed should be improved while maintaining diversity.

Therefore, the purpose of this study was to measure the degree of genomic inbreeding in Japanese Black cattle at a chromosomal level and use it to examine inbreeding depression in their reproductive ability.

## MATERIALS AND METHODS

Reproductive records, SNP information, and pedigree information of Japanese Black cattle born between February 2001 and July 2005 were collected from large-scale collective farms at Tochigi Prefecture. This large-scale collective farm has standardized its animal management manual. Reproductive records that had fewer than ten animals per farm were removed. Accordingly, 8,553 reproductive records were obtained. Genomic inbreeding was analyzed using SNP information from 782 animals selected from the total records. First, the fixed effects on reproductive traits were estimated using pedigree information of 8,553 animals. Phenotypic values corrected for fixed effects were then used to estimate inbreeding depression.

The analyzed traits were age at first calving (AFC), a trait targeted by the Ministry of Agriculture, Forestry, and Fisheries in Japan to improve reproductive performance, and calf production index (CPI), an overall indicator of reproductive performance. The CPI is the number of calves born before the first parturition after reaching the age of four and was converted to the value at the age of four. CPI was calculated using the following formula:


(1) $$\text{CPI}=\frac{4-\text{AFC}}{\text{CI}}+1$$

Where, CI is calving interval (year).

A summary of each trait is presented in Table 1.

To analyze inbreeding depression, the phenotypic values were adjusted using fixed effects. In 782 animals, regressions of adjusted phenotypic values on genomic inbreeding were estimated for the whole genome, each chromosome, and significant chromosomal segment regions. The difference of the estimated regression coefficients from zero was tested with Bonferroni correction.

We used the following statistical model to estimate each fixed effect.


(2) $$y_{ijk}=\mu +a_{ijk}+Farm_{i}+By_{j}+e_{ijk}$$

Where *y_ijk_* is the phenotypic value, μ is the population mean, *a_ijk_* is the breeding value (random effect), *Farm_i_* is the farm (118 levels) (fixed effect), *By_j_* is the birth year and month (54 levels) (fixed effect), and *e_ijk_* is the residual (random effect). Pedigree information was traced back two generations. The THRGIBBS1F90 program [4] of the BLUPF90 program family was used for the analysis, and Bayesian estimation was performed using Gibbs sampling method. In the Gibbs Sampling method, 1,000,000 samples were taken, with a burn-in set to 500,000 cycles, spacing set to 50 cycles, and a total of 10,000 samples obtained. The mean of 10,000 samples was used as the estimate. Although breeding values were estimated, they were not used in the analysis. Only estimates of fixed effects were used.

SNPs with a minor allele frequency less than 0.01 and a call rate less than 0.95 were removed, resulting in 37,129 SNPs per individual. PLINK [5] was used to measure the ROH. PLINK detects the ROH using a window approach with multiple ROH settings. In this study, we used three different settings according to Martikainen et al (Table 2) [6].

Based on the following formula, the ratio of the measured ROH to the whole genome was defined as the degree of genomic inbreeding (F_ROH_). F_ROH_ was calculated at the whole-genome level and chromosomal level.


(3) $${\text{F}}_{\text{ROH}=}\Sigma {\text{L}}_{\text{ROH}}/{\text{L}}_{\text{auto}}$$

ΣL_ROH_ is the total ROH length in the whole genome (bp), and L_auto_ is the total length of the whole autosomal genome (bp). F_ROH1_, F_ROH2_, and F_ROH3_ were measured according to ROH definitions 1 to 3, respectively. For chromosomes that showed significant inbreeding depression during analysis, the degree of genomic inbreeding and inbreeding depression were analyzed in the segmented regions of the chromosome. The division method has been described by Martikainen et al [7]. First, half the length of the entire region of each chromosome was considered the measurement region. Next, the region to be measured was shifted across the chromosome by half its length, and F_ROH_ was measured in each region. Subsequently, regression of the corrected phenotypic value of F_ROH_ was estimated and used to determine the extent of inbreeding depression. This method was repeated thrice for each chromosome. Pedigree information has also been used to measure conventional inbreeding coefficients. The pedigree information included 496,739 animals. The inbreeding coefficients were determined by tracing two generations: 5 and 22, denoted as F_PED5 and_ F_PED22,_ respectively.

## RESULTS

Table 3 shows a summary of the genomic inbreeding degree and the inbreeding coefficient. The mean of genomic inbreeding was higher than that of inbreeding coefficient. Although there were animals with an inbreeding coefficient of 0, none had F_ROH1_ or F_ROH2_ of 0. This indicated that all animals include ROH in these two settings. The average F_ROH2_ was higher than that of F_ROH1_ and F_ROH3_ because many short ROHs of 5 Mbp or less were detected in the definition of F_ROH 2_.

Table 4 shows the correlation between the degree of genomic inbreeding and the inbreeding coefficient, with a high positive correlation observed. Overall, the correlation between the degree of genomic inbreeding and coefficient of inbreeding was moderately positive (0.55–0.61). Additionally, the correlation between genomic inbreeding and F_PED22_ was almost the same with ROH definitions 1, 2, and 3. The correlation between F_ROH 2_ and F_PED22_ was slightly higher than that between F_ROH 2_ and F_PED22_ due to the detection of small ROH, reflecting earlier generations.

Table 5 shows the regression and standardized regression coefficients of inbreeding on reproductive traits. In CPI, significant inbreeding depression was detected at all genomic inbreeding degrees (F_ROH1,_ F_ROH2,_ F_ROH3_: −0.011, −0.011, −0.010, respectively). However, no significant difference was observed in the inbreeding coefficient. No significant inbreeding depression was detected for either genomic inbreeding or inbreeding coefficient in AFC. The heritability of CPI and ACF was 0.19 and 0.18, respectively, indicating a slightly low heritability. Therefore, the lack of inbreeding depression may be due to a stronger environmental influence or limited genetic variance.

F_ROH 1_, F_ROH 2_, and F_ROH 3_ were calculated on chromosomes 1–29. The means for each chromosome were 0.00–0.079, 0.03–0.13, and 0.001–0.067 for F_ROH1_, F_ROH2_, and F_ROH3_, respectively. Given that ROH was not detected on chromosomes 24 and 26 of F_ROH 1_, the degree of genomic inbreeding was 0. No particular trend was observed in the frequency of ROH on other chromosomes. F_ROH 2_ was higher than F_ROH 1_ and F_ROH 3_, as well as the genomic inbreeding of the whole genome.

Table 6 shows the regression coefficients of CPI and AFC for chromosomes with significant inbreeding depression at chromosome level. In CPI, significant inbreeding depression common to F_ROH 1_, F_ROH 2_, and F_ROH 3_ was identified on chromosomes 12, 15, and 27, respectively. In AFC, significant inbreeding depression common to F_ROH1_, F_ROH2_, and F_ROH3_ was identified on chromosome 4. Significant inbreeding depression was observed on chromosome 4 (F_ROH2_: −0.003) in CPI, chromosome 12 (F_ROH2_: 0.9), and chromosomes 27 (F_ROH1_: 0.7) in AFC.

Table 7 shows the standardized regression coefficients used to compare the extent of significant inbreeding depression at a chromosome level. In CPI, the highest standardized regression coefficient was in chromosome 15 (F_ROH2_: −0.12). In AFC, the highest standardized regression coefficient was in chromosome 4 (F_ROH2_: 0.11).

In subsequent studies of intrachromosomal regions, chromosomes 12, 15, and, 27 (CPI) and chromosome 4 (AFC) were analyzed because they were significant in inbreeding depression. The degree of genomic inbreeding and the regression coefficient of each region were analyzed.

On chromosome 12, inbreeding depression was significantly detected in the regions 45545800 to 91091600 of F_ROH2_ in division 1, and 34159350 to 56932250 of F_ROH3_ and 68318700 to 91091600 of F_ROH2_ in division 2. No inbreeding depression was detected in division 3.

On chromosome 15, significant inbreeding depression was detected in the regions 21314328 to 63942984 and 42628656 to 85257312 in division 1, 42628656 to 63942984 in division 2, and 53285820 to 63942984 in division 3. In addition, significant inbreeding depression was detected in the regions 21314328 to 42628656 of F_ROH2_ in division 2 and 47957238 to 58614402 of F_ROH1_ and F_ROH2_ in division 3.

On chromosome 27, significant inbreeding degeneration was detected in the regions 11342247 to 34026740 in division 1 and 22684494 to 28355617 in division 3 for F_ROH1_, F_ROH 2_, and F_ROH3._ In addition, significant inbreeding degeneration was detected in the regions 17013370 to 28355617 of F_ROH2_ and F_ROH3_ in division 2, and 22684494 to 34026740 of F_ROH2_ and 19848932 to 25520055 of F_ROH2_ in division 3.

On chromosome 4, significant inbreeding depression was detected in the regions 0 to 60307635 of F_ROH 1_, F_ROH 2_, and F_ROH 3_ in division 1. In addition, significant inbreeding depression was detected in the regions 30153817 to 90461452 in division 1, 0 to 30153817 in division 2, and 45230726 to 75384543 in division 2.

Table 8 shows the significant regression coefficients and the number of genes in each region. The shorter the region, the fewer the genes it contains.

LOC787432 (follitropin subunit beta) and FSHB (follitropin subunit beta precursor) were found to be involved in reproduction in the effective region 53285820 to 63942984 on chromosome 15 (Table 9).

No genes directly related to reproduction were found in the significant region 22684494 to 28355616 on chromosome 27.

## DISCUSSION

Compared with the inbreeding coefficient, degree of genomic inbreeding was higher, suggesting that ROH enables the detection of homozygous regions that cannot be measured by conventional inbreeding coefficients.

The inbreeding coefficient is estimated using pedigree information. Therefore, depending on the existence pattern of common ancestors, some individuals may have the same inbreeding coefficient value. Conversely, the degree of genomic inbreeding, estimated using ROH, shows a larger variation. Therefore, the distribution of the degree of genomic inbreeding is close to a normal distribution, whereas the distribution of the inbreeding coefficient is highly kurtotic, as the inbreeding coefficient was 0 in many animals.

F_ROH2_ had a higher value than F_ROH1_ and F_ROH3_ because many short ROHs of 5 Mbp or less were detected in F_ROH 2_. Similar results were reported by Martikainen et al [6] who defined ROH.

The inbreeding coefficient and the degree of genomic inbreeding showed a moderate correlation of 0.55 and 0.61. Similar results have been reported in a previous study. Sumreddee et al [8] reported a correlation of 0.661 between the degree of genomic inbreeding and the inbreeding coefficient in Hereford cattle. In addition, Pryce et al [9] reported a correlation of 0.53 for Holstein and 0.51 for Jersey. Therefore, the degree of genomic inbreeding and inbreeding coefficient have a moderate correlation, and the degree of genomic inbreeding can somewhat explain the conventional inbreeding coefficient.

Inbreeding depression was only significant in the CPI for genomic inbreeding. Several studies investigated inbreeding depression in fertility using genomic inbreeding. Bjelland et al [10] showed that a 1% increase in F_ROH_ in Holstein breeds changed days open by 1.72 days, conception rate by −0.82%, and calving ease (5-point scale) by 0.03. Similarly, Martikainen et al [6] showed that inbreeding depression occurred during the interval from calving to first insemination and interval from first to last insemination in Ayrshire cattle using F_ROH_. The population used in this study also experienced inbreeding depression in the same manner.

The genomic inbreeding F_ROH1_, F_ROH2,_ F_ROH3_ of all chromosomes were 0.059, 0.109, and 0.050, respectively. Almost of genomic inbreeding of each chromosome did not differ significantly from that of whole chromosomes, however F_ROH1_ of chromosomes 24 and 26 was 0. Chromosomes 24 and 26 showed large differences in the definition of ROH. Although there are many homozygous SNPs in these chromosomes, their density is not high and they are located far apart. Therefore, ROH could not be detected in ROH1, defined by density, and ROH was detected in ROH2 and ROH3, where density is not considered.

Inbreeding depression was detected on chromosomes 12, 15, and 27 for CPI and chromosome 4 for AFC, in line with F_ROH 1_, F_ROH 2_, and F_ROH3_, respectively_._ In a study using F_ROH_, Martikainen et al [7] detected an inbreeding depression on chromosomes 2, 15, 18, and 22 for fertility traits. In a study using ROH, Pryce et al [9] reported that Holsteins have ROHs on chromosomes 2, 5, 8, 9, 15, and 24 that are effective for reproductive ability. Although only chromosome 15 is consistent across these studies, it is highly possible that many chromosomes are different because inbreeding depression occurs in a population- and breed-specific manner.

From the standardized regression coefficient results, the magnitude of inbreeding depression in the CPI for this population decreases in the order of chromosomes 15, 12, and 27. This suggests that the ROH region on chromosome 15 retains more genes that affect the CPI than the ROH regions on other chromosomes.

When comparing the CPI with AFC, the CPI detected more significant inbreeding depression. This difference is due to the measurement method of the two indicators. AFC is the number of days until the first delivery after birth. However, CPI is a composite index that uses AFC and calving interval. The difference in the number of chromosomes with inbreeding depression in the CPI and AFC might be due to the calving interval.

Significant inbreeding depression was detected in division 3 on chromosomes 15 and 27 and in division 2 on chromosomes 4 and 12. The significant regions in division 3 were included in the significant regions in division 2, and the significant regions in division 2 were included in the significant regions in division 1. From these results, it may be assumed that the inbreeding depression region in division 1 is caused by inbreeding depression in division 2, and that the inbreeding depression region in division 2 is also caused by inbreeding depression in division 3. However, no significant inbreeding depression regions were detected in division 3 on chromosomes 4 and 12. This may be because the effect of genes on traits is small, and genes are widely scattered; therefore, the region could not be identified. Whereas on chromosomes 15 and 27, the genes that have an effect are concentrated in the division 3 region and genes with a larger effect are present.

On chromosomes 4 and 12, many inbreeding depression regions were detected, which were not common across ROH definitions 1, 2, and 3. Regions showing such inbreeding depression depend on the ROH definition used. It is necessary to perform the appropriate ROH definition and gain a structural understanding of the detected ROH.

Two genes on chromosome 15, LOC787432 and FSHB, are reproduction-related genes. Therefore, it is suggested that the region from 53285820 to 63942984 on chromosome 15 is the causative gene for inbreeding depression. Genetic mutations in the 5’ upstream regulatory region of FSHB are associated with decreased serum follicle-stimulating hormone levels, semen quality, and fertility in bulls [11]. Therefore, it is highly likely that some functions are impaired in calves.

No genes directly related to reproduction were found in the region 22684494 to 28355616 on chromosome 27. It is conceivable that existing genes indirectly affect fertility; however, the existence of unknown genes has yet to be identified.

The genomic inbreeding coefficient of the population used in this study was higher than the inbreeding coefficient, with a moderate correlation between the two. In addition, inbreeding depression due to genomic inbreeding was detected using the CPI and AFC. Therefore, the degree of genomic inbreeding is effective in detecting inbreeding depression in the reproductive ability of Japanese Black cattle. In addition, inbreeding depression was detected on chromosomes 12, 15, and 27 for CPI and on chromosome 4 for AFC. Therefore, the chromosome responsible for inbreeding depression was identified. A specific region of approximately 1/8 the length of chromosomes 15 and 27 showed inbreeding depression in the CPI.

## CONCLUSION

These results suggest that mating that takes the degree of genomic inbreeding into consideration is effective in decreasing the degree of inbreeding, and considering the degree of genomic inbreeding is effective in improving the reproductive ability of Japanese Black cattle. Genomic inbreeding has the potential to be an effective approach for maintaining diversity and improving the reproductive capacity of this breed.

## Figures and Tables

**Table 1:** Summary of reproductive trait

Population	Trait	Mean	SD	Min	Max	N
Whole	CPI	2.58	0.343	1.48	3.48	8,553
	AFC	836.7	104.108	614	1,124	8,553
With SNPs information	CPI	2.46	0.520	1.48	3.48	782
	AFC	866.3	135.043	626	1,124	782

SD, standard deviation; CPI, calf production index; AFC, age at first calving; SNP, single nucleotide polymorphism.

**Table 2:** Definition of ROH setting

ROH setting	SNP density (kb)	Window size (kb)	Number of SNPs
1	≥1/120	≥500	-
2	≥1/1,000	≥10	≥30
3	≥1/1,000	≥10	≥100

ROH, run of homozygosity; SNP, single nucleotide polymorphism.

**Table 3:** Summary of the genomic inbreeding degree and inbreeding coefficient

	Mean	SD	Min	Max	CV
F_ROH1_	0.059	0.040	0.012	0.25	0.68
F_ROH2_	0.109	0.044	0.032	0.30	0.40
F_ROH3_	0.050	0.039	0	0.25	0.78
F_PED5_	0.023	0.028	0	0.16	1.22
F_PED22_	0.036	0.031	0	0.17	0.87

SD, standard deviation; CV, coefficient of variation.

**Table 4:** Correlation between the degree of genomic inbreeding and the inbreeding coefficient

	F_ROH1_	F_ROH2_	F_ROH3_	F_PED5_	F_PED22_
F_ROH1_	1				
F_ROH2_	0.97	1			
F_ROH3_	0.99	0.97	1		
F_PED5_	0.59	0.55	0.59	1	
F_PED22_	0.61	0.60	0.61	0.95	1

**Table 5:** Regression and standardized regression coefficients of inbreeding on reproductive traits

	CPI		AFC	
	
	Regression	Standardized regression	Regression	Standardized regression
F_ROH1_	−0.011^*^	−0.083^*^	1.79	0.055
F_ROH2_	−0.011^*^	−0.095^*^	2.01	0.068
F_ROH3_	−0.010^*^	−0.078^*^	1.55	0.047
F_PED5_	−0.0038	−0.021	1.09	0.024
F_PED22_	−0.0065	−0.041	1.91	0.047

* p<0.05.

CPI, calf production index; AFC, age at first calving.

**Table 6:** Regression coefficients for calf production index and age at first calving for each chromosome

Chromosome No.	CPI			AFC		
	
	F_ROH1_	F_ROH2_	F_ROH3_	F_ROH1_	F_ROH2_	F_ROH3_
4	−0.003	−0.003^*^	−0.002	1.0^**^	1.1^**^	0.9^*^
12	−0.004^*^	−0.004^**^	−0.004^*^	0.7	0.9^*^	0.7
15	−0.004^**^	−0.004^**^	−0.004^**^	0.5	0.5	0.5
27	−0.003^*^	−0.003^*^	−0.003^*^	0.7^*^	0.6	0.7

Regression coefficients were estimated against the genomic inbreeding (%).

* p<0.05,

** p<0.01.

CPI, calf production index; AFC, age at first calving.

**Table 7:** Standardized regression coefficients for calf production index and age at first calving for each chromosome

Chromosome No.	CPI			AFC		
	
	F_ROH1_	F_ROH2_	F_ROH3_	F_ROH1_	F_ROH2_	F_ROH3_
4	−0.07	−0.07^*^	−0.06	0.10^**^	0.11^**^	0.09^*^
12	−0.09^*^	−0.10^**^	−0.08^*^	0.07	0.08^*^	0.06
15	−0.11^**^	−0.12^**^	−0.11^**^	0.05	0.05	0.05
27	−0.08^*^	−0.07^*^	−0.08^*^	0.07^*^	0.06	0.06

* p<0.05,

** p<0.01.

CPI, calf production index; AFC, age at first calving.

**Table 8:** Regions where significant regression coefficients were detected and the number of genes in each region

Chromosome	No of division	Start (bp)	End (bp)	ROH definition	Number of genes
Calf production index					
12	1	45545800	91091600	2	415
12	2	68318700	91091600	2	270
12	2	34159350	56932250	3	235
15	1	21314328	63942984	1, 2, 3	1,351
15	1	42628656	85257312	1, 2, 3	1,547
15	2	21314328	42628656	1	565
15	2	42628656	63942984	1, 2, 3	795
15	3	47957238	58614402	1, 2	458
15	3	53285820	63942984	1, 2, 3	251
27	1	11342247	34026740.3	1, 2, 3	337
27	2	17013370	28355616.9	2, 3	274
27	2	22684494	34026740.3	2	160
27	3	19848932	25520055.2	2	52
27	3	22684494	28355616.9	1, 2, 3	69
Age at first calving					
4	1	0	60307634	1, 2, 3	811
4	1	30153817	90461451	1, 2	924
4	2	0	30153817	2	368
4	2	45230726	75384543	1, 2	491

ROH, run of homozygosity.

**Table 9:** Genes associated with fertility in the effective region of chromosome 15

Gene	Protein	Start	End	Length (kbp)
*LOC787432*	Follitropin subunit beta	60911442	60917442	128
*FSHB*	Follitropin subunit beta precursor	60977654	60979618	129
